# The long-term effects of meteorological parameters on pertussis infections in Chongqing, China, 2004–2018

**DOI:** 10.1038/s41598-020-74363-8

**Published:** 2020-10-14

**Authors:** Yongbin Wang, Chunjie Xu, Jingchao Ren, Yingzheng Zhao, Yuchun Li, Lei Wang, Sanqiao Yao

**Affiliations:** 1 Department of Epidemiology and Health Statistics, School of Public Health, Xinxiang Medical University, Henan Province, Xinxiang, 453000 People’s Republic of China; 2 Department of Occupational and Environmental Health, School of Public Health, Capital Medical University, Beijing, People’s Republic of China; 3 Center for Musculoskeletal Surgery, Charité–Universitätsmedizin Berlin, Corporate Member of Freie Universität Berlin, Humboldt–Universität Zu Berlin and Berlin Institute of Health, Berlin, Germany

**Keywords:** Diseases, Infectious diseases, Medical research, Epidemiology

## Abstract

Evidence on the long-term influence of climatic variables on pertussis is limited. This study aims to explore the long-term quantitative relationship between weather variability and pertussis. Data on the monthly number of pertussis cases and weather parameters in Chongqing in the period of 2004–2018 were collected. Then, we used a negative binomial multivariable regression model and cointegration testing to examine the association of variations in monthly meteorological parameters and pertussis. Descriptive statistics exhibited that the pertussis incidence rose from 0.251 per 100,000 people in 2004 to 3.661 per 100,000 persons in 2018, and pertussis was a seasonal illness, peaked in spring and summer. The results from the regression model that allowed for the long-term trends, seasonality, autoregression, and delayed effects after correcting for overdispersion showed that a 1 hPa increment in the delayed one-month air pressure contributed to a 3.559% (95% CI 0.746–6.293%) reduction in the monthly number of pertussis cases; a 10 mm increment in the monthly aggregate precipitation, a 1 °C increment in the monthly average temperature, and a 1 m/s increment in the monthly average wind velocity resulted in 3.641% (95% CI 0.960–6.330%), 19.496% (95% CI 2.368–39.490%), and 3.812 (95% CI 1.243–11.690)-fold increases in the monthly number of pertussis cases, respectively. The roles of the mentioned weather parameters in the transmission of pertussis were also evidenced by a sensitivity analysis. The cointegration testing suggested a significant value among variables. Climatic factors, particularly monthly temperature, precipitation, air pressure, and wind velocity, play a role in the transmission of pertussis. This finding will be of great help in understanding the epidemic trends of pertussis in the future, and weather variability should be taken into account in the prevention and control of pertussis.

## Introduction

Pertussis, also known as 100-day cough, is a contagious illness of the respiratory tract caused by *Bordetella pertussis* that mainly affects children at the age of 1–6 years, though other ages can also be involved^1,2^. The disease is easily transmitted from children to children through the droplets as a result of coughing or sneezing of the people contracting pertussis^3^. Before the nineteenth century, this disease was one of the major causes of incidence and death among children in the world^4^. However, the pertussis morbidity had a noticeable decrease with the introduction of the vaccine available since the twentieth century^3,5^. It was estimated that global vaccination against this disease prevented about 687,000 deaths in 2008^6^. Currently, the proportion of the susceptible population vaccinated with the well-established vaccine in 2018 has reached 86% and 99% all over the world and in China, respectively^6,7^. But, several countries and areas are experiencing a re-emerging risk in the incidence of pertussis in recent years, such as Amhara regional state, mainland China, Italy, France, India, Canada and the like^3,8–10^. In 2019, there were 132,754 cases reported on the global scale, and around 95% of which were recorded in developing countries^6^. Among them having the most reported cases is China with the numbers of 30,027 notified in 2019, accounting for 22.62% of the totals worldwide^6^. Importantly, several epidemiological studies have suggested that the morbidity of pertussis may be seriously underreported to a large extent in China^11–13^. Furthermore, escalating work is showing that there is a tendency to continue to increase in the incidence of pertussis in China^1,3,14^. However, little is so far known regarding the causes of this vaccine-preventable disease in its growing numbers. Hence, investigating its potential drivers and forming prevention and control planning has become critical and urgent for tackling such an increasing threat.

In recent years, the relationship between weather variability and contagious diseases has received increasingly wide attention, because climatic drivers may play a pivotal role in the dynamics, distribution, and transmission of contagious diseases via impacting the growth and development of pathogenic agents, population dynamics of hosts and human behaviors, and thus can be deemed as early warning signals for the dynamic epidemics of contagious diseases^15,16^. Presently, many publications have found that meteorological parameters are closely related to the incidences of scarlet fever^17^, tuberculosis^18^, dengue fever^19^, hemorrhagic fever with renal syndrome (HFRS)^20^, bacillary dysentery^21^, human brucellosis^22^, hand, foot and mouth disease (HFMD)^23^, etc. Prior studies also reported the relationship between weather parameters and pertussis. For example, Huang et al. found that a 1 °C increment in the monthly mean minimum temperature was correlated with a 3.1% (95% CI 1.3–4.8%) decrease in monthly pertussis morbidity^24^. Blackwood et al. reported that the rainy season may play an important role in driving the seasonal pattern of pertussis transmission^25^. Zhang et al. found that mean temperature and relative humidity may increase the incidence risk of pertussis among different age groups^14,26^. However, these researches only assessed the influences of one or two meteorological parameters on pertussis. A deeper understanding of the long-term role of weather variability during the spread of diseases offers a basis for forecasting the health effects of global climate change^27^, and given that most of the respiratory infectious diseases have also been confirmed to be correlated with wind speed, sunshine, atmospheric pressure, and precipitation^22,28,29^. Thus, it is necessary to conduct a study that simultaneously considered common weather parameters (e.g. temperature, relative humidity, wind speed, sunshine, atmospheric pressure, and precipitation) to synthetically elucidate the long-term quantitative association between meteorological factors and pertussis, in order to address the increasing threat posed by pertussis to public health.

Chongqing, located in the Three Gorges Reservoir Area, is the only municipality in Southwest China. It has been experiencing a tremendous natural change owing to the establishment of the Three Gorges Dam, which is the largest hydroelectric station worldwide. In recent decades, Chongqing is among the highest-risk areas of pertussis in China (Fig. S1). As far as we are aware, there is so far no literature on whether the climatic factors are associated with the resurgence of pertussis in this city. Here, we performed a time series analysis to explore the long-run impact of meteorological drivers on pertussis.

## Material and methods

### Data collection

In this research, data on the monthly incidents of pertussis in Chongqing city from January 1, 2004 through December 31, 2018 were supplied by the Chinese Center for Disease Control and Prevention (CDC), the population numbers from the same period were extracted from the website of Chongqing Statistics (https://tjj.cq.gov.cn/tjsj/sjjd/) (Fig. S2). All notified cases were diagnosed based on the criteria for notifiable infectious diseases in China, and the detailed description regarding this diagnostic guideline has been released by the National Health Commission of the people's Republic of China in 2007^30^.

Meteorological data, including average temperature (°C), average atmospheric pressure (hPa), aggregate precipitation (mm), aggregate sunshine hours (h), average relative humidity (%), and average wind velocity (m/s), were offered by the National Meteorological Science Center. Then we assembled all these variables in a monthly format (Figs. S3–S8).

### Ethics statement

The study protocol was approved by the research institutional review board of the Xinxiang Medical University (No: XYLL-2019072), and the need of informed consent was waived by the ethics committee because all the data used in our work were collected in an anonymous way and we cannot access to any personal identifying information with an exception of the publicly available reported counts. This research met all the guidelines outlined in the Declaration of Helsinki.

### Statistical analysis

In the statistical description, the incidence time series of pertussis and climatic factors were represented as mean ± standard deviation ($\overline{x} \pm s$) or interquartile range (IQR). Then spearman's correlation coefficient matrix was employed to analyze the correlation between climatic variables and pertussis. Considering that the incidence data of infectious diseases are often over-dispersed and the relationship between weather parameters and pertussis cases tends to be linear in Chongqing, 2004–2018 (Fig. S9)^20^, we thus used a negative binomial multivariable regression to assess the independent effect of variations in monthly meteorological factors on the morbidity of pertussis. In this multivariable regression analysis, given that the climatic drivers for the months prior to the occurrence of infectious diseases are frequently critical^20,22,31,32^, and that the majority of infected people often have a mean incubation period ranging from 2 to 21 days (but some patients’ symptoms can last for 1–2 months^33^) and there is a delay of up to 2 weeks from the confirmed cases to the Statutory Infectious Disease Reporting System^14^, so we included climatic factors with a lag of 0–2 months in our regression model. Meanwhile, given that the multicollinearity among the covariates can lead to unstable estimated parameters, hence we used the variance inflation factor (VIF) to evaluate the degree of multicollinearity^34^. If the VIF value was to exceed 10, it could suggest a strong co-linearity^34^. In this case, these two drivers failed to be put together into the regression. Instead, they should be put separately with other meteorological factors to estimate their independent contribution to pertussis in different models. The basic representation of the regression model is expressed as

1 $$ \log \, (\hat{Y}_{t} ) = \beta_{0} + \beta_{1} X_{t1} + \beta_{2} X_{t2} + \cdots + \beta_{n} X_{tn} + \beta_{m} \;{\text{month}}_{t} + \beta_{y} \;{\text{year}}_{t} + \log k_{t} $$

where ${\hat{\text{Y}}}_{{\text{t}}}$ is the expected number of pertussis cases at time t (for infectious diseases, the count variable is overdispersed), $\beta_{0}$ signifies intercept,$\beta_{1} ,\beta_{2} , \cdots ,\beta_{n}$ are the estimated coefficients of the monthly climatic drivers $X_{t1} ,X_{t2} \cdots X_{tn}$, $\beta_{m}$ and $\beta_{y}$ represent the estimated coefficients of the month and year variables at time t, respectively, and $k_{t}$ is the dispersion degree which means that there exists a clustered phenomenon of the data, namely, overdispersion in Poisson models occurs as pertussis is characterized by infection, leading to the violations of the likelihood independence of observations assumption. At this time, the response variance is often greater than the mean in the data (namely, the counts follow a Poisson–gamma mixture distribution)^35^. The negative binomial regression model is based on the requirement that counts are dependent on one another, and it is derived from a Poisson–gamma mixture distribution. In the course of analysis, first, the month (dummy variables) and year variables were incorporated into the regression model to remove the impacts of the seasonal effects and long-run trends. Second, the autocorrelation between pertussis cases was further considered in the final regression model (the one with the variables of lags has a larger effect and more meteorological parameters with statistical significance as compared to others should be deemed as the final regression model). The partial autocorrelation function (PACF) plot is able to describe the correlation between the pertussis cases and its past cases under the condition of given cases, and hence which was used to determine the orders of the autocorrelation^36^. The cointegration testing of the cointegrating regression under the Engle-Granger test can be used to characterize the long-run relationship between the variables^37^. Hence the stationarity and the long-run relationship between pertussis and meteorological parameters were further tested with the Augmented Dickey-Fuller (ADF) and cointegration analyses in order to avoid producing misleading results owing to pseudo-regression^37^. A standardized change (z-score) was employed to remove the dimensions of different variables.

2 $$ {\text{z } - \text{ score}} = \frac{{X_{i} - \overline{X}}}{S} $$

where *X*_*i*_ denotes the original values of all variables, $\overline{X}$ is the average of the original values, and *S* signifies the standard deviation of the original values. By doing so, the mean of the resulting data series is 0, the variance is 1, and the dimension was removed from all the actual variables.

Additionally, a sensitivity analysis with a 2-month moving average lag for the weather variables was performed to validate the stability of our study results. Incident rate ratio (IRR) with its corresponding 95% confidence intervals (95% CI) were computed based on the regression coefficients (namely, equal to exp($\beta$)) to assess the independent contribution of meteorological factors to the incidence of pertussis. In this study, the regression model was developed with SPSS software (version 24.0, IBM Corp, Armonk, NY), and the cointegration testing and all the plots were performed using Eviews10.0 software (IHS, Inc. USA).

## Results

### Statistical description

Over the study period of 2004–2018, the number of pertussis cases total 3,871 in Chongqing city, leading to an annualized incidence of 0.781 per 100,000 population. The pertussis incidence rose from 0.251 per 100,000 people in 2004 to 3.661 per 100,000 persons in 2018, with a drastic increase of 14.586 folds. There were noticeable seasonal patterns in the morbidity of pertussis, peaked in March to August per year, with the highest peak occurred in July; whereas a trough was observed in September until February of the subsequent year (Fig. S10).

The distribution characteristics of the meteorological variables are summarized in Table 1, displaying that the monthly averages of aggregate precipitation, temperature, aggregate sunshine hours, relative humidity, wind velocity, and air pressure were 80.700 (31.425, 126.900), 19.300 (12.500, 24.675), 64.800 (33.200, 254.300), 78.300 (72.550, 90.000), 1.400 (1.300, 1.500), and 979.804 ± 9.095, respectively.

**Table 1:** Summary statistics for the monthly pertussis cases and weather parameters in Chongqing, China, 2004–2018.

Variable	Mean	S.D	Min	P_25_	P_50_	P_75_	Max
Pertussis cases	21.510	37.718	0.000	1.000	4.500	19.750	190.000
AP (mm)	96.696	82.852	3.400	31.425	80.700	126.900	553.400
AT ( °C)	18.694	7.340	3.900	12.500	19.300	24.675	32.400
ASH (h)	81.831	62.545	0.000	33.200	64.800	119.825	254.300
ARH (%)	77.259	7.202	49.000	72.550	78.300	83.000	90.000
AWV (m/s)	1.406	0.208	0.900	1.300	1.400	1.500	2.000
AAP (hPa)	979.804	9.095	956.400	973.750	979.688	987.675	996.100

*AP* aggregate precipitation, *AT* Average temperature, *ASH* aggregate sunshine hours, *ARH* average relative humidity, *AWV* average wind velocity, *AAP* average air pressure, *S.D.* standard deviation.

### Correlation analysis

**Figure 1:**
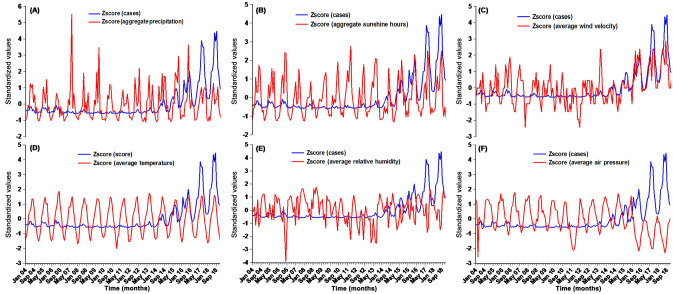
Time series plot displaying the monthly pertussis incidence and six climatic variables after standardized transformation. (**A**) Aggregate precipitation; (**B**) Aggregate sunshine hours; (**C**) Average wind velocity; (**D**) Average temperature; (**E**) Average relative humidity; (**F**) Average air pressure.

Time series displaying the monthly number of pertussis cases and climatic variables are presented in Fig. 1. In view of the different dimensions, we conducted a standardized change (z-score) to remove the dimensions of all variables. We observed that the monthly pertussis cases showed a similar temporal trend as the aggregate precipitation, average temperature, aggregate sunshine hours, and average wind velocity. The mentioned-above meteorological variables are positively in relation to the monthly pertussis cases. While a contrary time trend was found between the pertussis cases and the monthly average relative humidity as well as the monthly average air pressure. The higher levels of these two factors were corresponding to a decrease in the reported cases. These findings were wholly aligned with the results originating from the spearman's correlation coefficient matrix (Table 2). Besides, the absence of correlation coefficients greater than 0.85 between meteorological variables indicated that no strong multicollinearity was present^20^, which was also evidenced by the collinearity statistics, as there were no VIF values greater than 10 when all variables were included into the regression model (Table 2).

**Table 2:** Spearman’s correlation coefficient matrix between variables and the collinearity statistics.

Variable	AP	AT	ASH	ARH	AWV	AAP	VIF
AP (mm)	1						2.017
AT ( °C)	0.734**	1					4.854
ASH (h)	0.570**	0.825**	1				7.439
ARH (%)	− 0.140	− 0.476**	− 0.723**	1			3.085
AWV (m/s)	0.347**	0.488**	0.610**	− 0.487**	1		1.860
AAP (hPa)	− 0.628**	− 0.717**	− 0.731**	0.407**	− 0.596**	1	2.647
Pertussis cases	0.313**	0.262**	0.359**	− 0.236**	0.590**	− 0.542**	–
Pertussis cases, 1-month lag	0.193**	0.082	0.192**	− 0.149*	0.456**	− 0.415**	–
Pertussis cases, 2-month lag	0.089	− 0.117	0.011	− 0.049	0.365**	− 0.227**	–

*AP* aggregate precipitation, *AT* average temperature, *ASH* aggregate sunshine hours, *ARH* average relative humidity, *AWV* average wind velocity, *AAP* average air pressure, *VIF* variance inflation factor.

*p < 0.05.

**p < 0.01.

### Negative binomial regression

**Figure 2:**
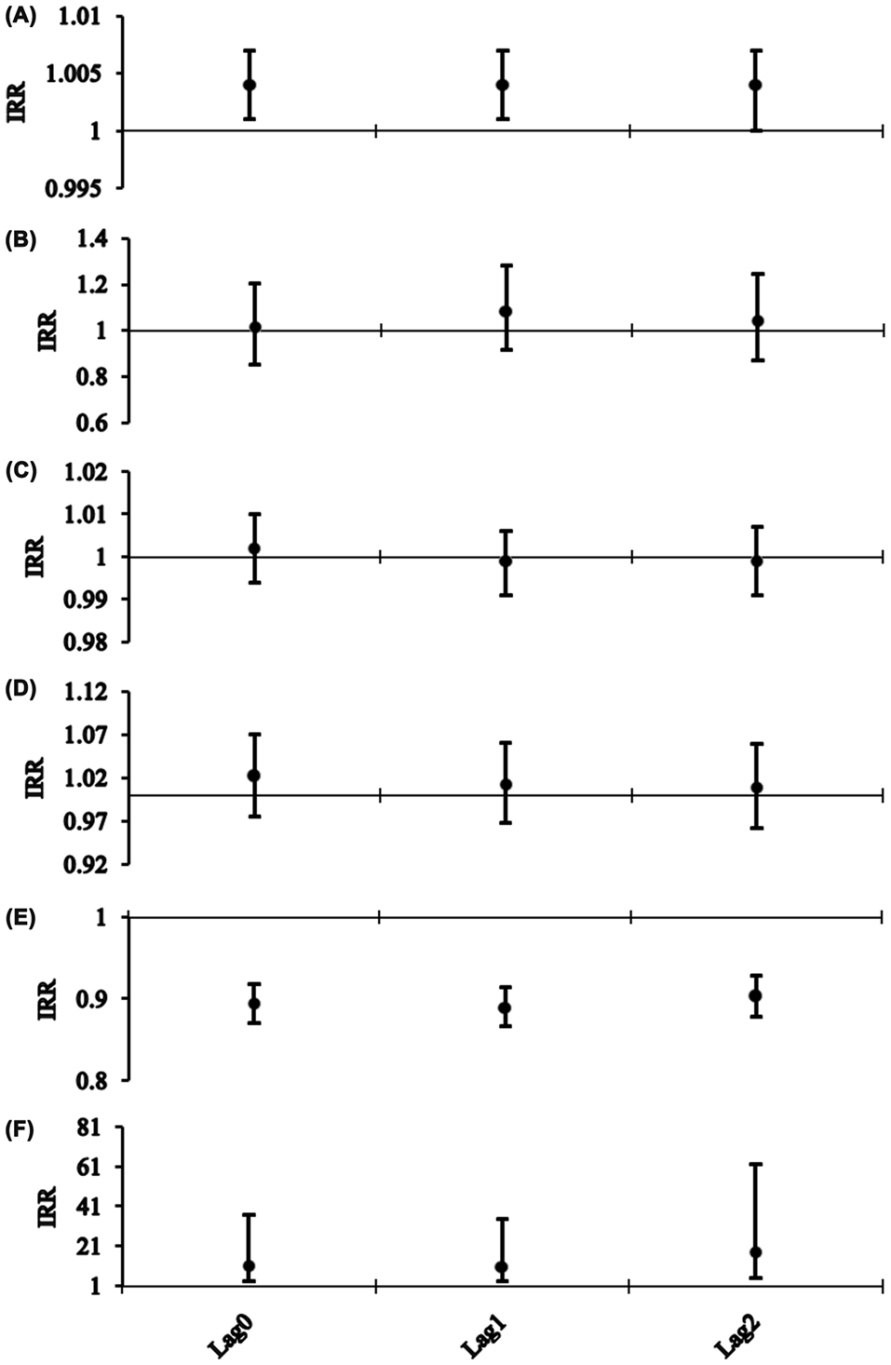
Negative binomial regression results of climatic variables correlated with the transmission of pertussis. (**A**) Aggregate precipitation; (**B**) Average temperature; (**C**) Aggregate sunshine hours; (**D**) Average relative humidity; (**E**) Average air pressure; (**F**) Average wind velocity.

**Figure 3:**
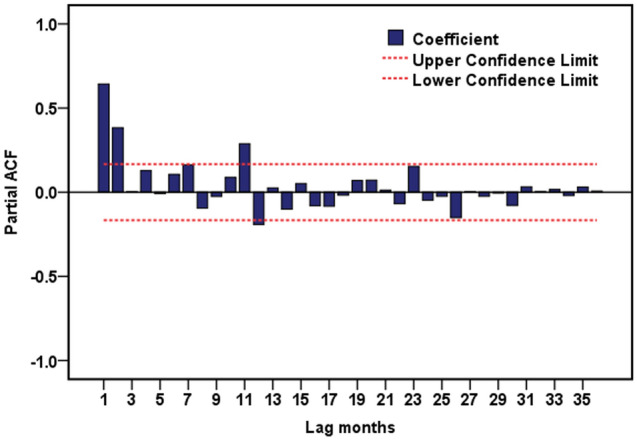
Partial autocorrelation function (PACF) plot for the seasonally differenced series. It was seen that there were two local maximum values at lag 1–2 months. So the autoregressive orders were considered to be 2.

**Figure 4:**
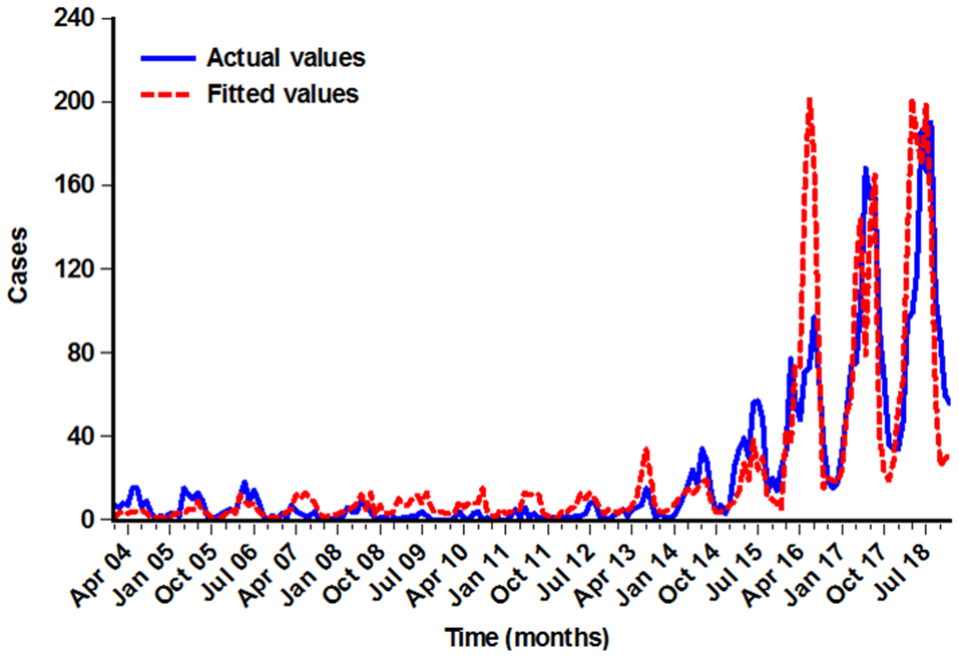
Comparison chart between the observed values and the fitted values based on the climatic variables.

In light of the delayed effects of climatic drivers on diseases, we thus constructed different regression models based on the lags of 0–2 months. Subsequently, we investigated the roles of meteorological factors played in the occurrence of pertussis by putting all the variables of different lags into above different models, because no obvious collinearity was detected among the predictors, and the results are given in Fig. 2 and Table S1, indicating noticeable delayed effects of meteorological factors on the monthly pertussis cases. After adjustment for the seasonality and long-term trends of pertussis incidence time series, aggregate precipitation, average wind velocity, and average air pressure at lags of 0–2 months were of significant relevance to the incidence of pertussis. The PACF plot seemingly suggested that the autoregressive orders should be considered to be 2 (Fig. 3). Further, the resulting results from the ultimate regression model that simultaneously allowed for the long-term trends, seasonality, autoregression (since the autoregressive order at lag 1 suggested no statistical difference when it was incorporated into the final model, the autoregressive order at lag 2 was only adjusted in the model), and delayed effects after correcting for overdispersion are given in Tables 3 and S2, showing that a 1 hPa increment in the delayed one-month air pressure might contribute to a 3.559% (95% CI 0.746–6.293%) decrease in the monthly number of pertussis cases; per 10 mm increment in the monthly aggregate precipitation, a 1 °C increment in the monthly average temperature, and a 1 m/s increment in the monthly average wind velocity might be associated with 3.641% (95% CI 0.960–6.330%), 19.496% (95% CI 2.368–39.490%), and 3.812 (95% CI 1.243–11.690)-fold increases in the monthly number of pertussis cases, respectively. The comparison between the fitted cases and the actual values in the ultimate regression model is illustrated in Fig. 4, and the resulting regression R value was 0.821 (this value measures the correlation between original observations and predicted values) (Fig. S11) and mean absolute error (MAE)^38^ was 11.094. Often, an R value greater than 0.8 means a relatively close relationship between observations and forecasts^39^.

**Table 3:** Estimated effects of meteorological parameters on pertussis morbidity by the final negative binomial regression.

Parameter	IRR	95% CI	*p*-value
AP (mm)* , 0-month lag	1.036	1.010–1.065	0.008
AT (°C), 0-month lag	1.195	1.024–1.395	0.024
ASH (h), 0-month lag	0.999	0.992–1.006	0.812
ARH (%), 0-month lag	1.008	0.968–1.050	0.702
AWV (m/s) , 0-month lag	3.812	1.243–11.690	0.019
AAP (hPa) , 1-month lag	0.964	0.937–0.993	0.014
Pertussis cases, 2-month lag	1.028	1.022–1.034	< 0.001

*IRR* incident rate ratio, *CI* confidence interval, *AP* aggregate precipitation, *AT* average temperature, *ASH* aggregate sunshine hours, *ARH* average relative humidity, *AWV* average wind velocity, *AAP* average air pressure.

*The effect of per 10 mm increment of aggregate precipitation on pertussis.

### Sensitivity analysis and cointegration testing

The resulting results from the sensitivity analysis with 2-month moving average lag for the weather variables are presented in Table 4, suggesting similar findings as the final regression model (i.e., the air pressure at 2-month moving average delay might contribute to decrease the number of pertussis cases, whereas the aggregate precipitation, the average temperature, and average wind velocity at 2-month moving average delay might be associated with the increases in the number of pertussis cases). The results of the ADF test statistics are summarized in Table 5, showing that the time series of pertussis incidence and above-identified four significant meteorological parameters was non-stationary, after differencing once, they became stationary. We thus performed cointegration testing based on these stationary series, the results are shown in Table 6, and the tau-statistics indicated *p*-values less than 0.05, which means that there may be a long-run equilibrium relationship between pertussis and climatic parameters. Also, this finding authenticates the results of the negative binomial regression analysis.

**Table 4:** Estimated effects of two-month moving averaged meteorological parameters on pertussis morbidity by the negative binomial regression in Chongqing, China, 2004–2018.

Parameter	IRR	95% CI	*p*-value
AP (mm)*, 2-month moving average lag	1.098	1.050–1.146	< 0.001
AT (°C), 2-month moving average lag	1.232	1.004–1.511	0.046
ASH (h), 2-month moving average lag	0.997	0.990–1.003	0.324
ARH (%), 2-month moving average lag	1.005	0.963–1.049	0.809
AWV (m/s) , 2-month moving average lag	7.572	1.830–31.333	0.005
AAP (hPa) , 2-month moving average lag	0.952	0.923–0.982	0.002
Pertussis cases, 2-month lag	1.022	1.016–1.029	< 0.001

*IRR* incident rate ratio, *CI* confidence interval, *AP* aggregate precipitation, *AT* average temperature, *ASH* aggregate sunshine hours, *ARH* average relative humidity, *AWV* average wind velocity, *AAP* average air pressure.

*The effect of per 10 mm increment of aggregate precipitation on pertussis.

**Table 5:** ADF test statistics for the pertussis cases and climatic parameters.

Variable	t-statistic	*p*-value	Variable	t-statistic	*p*-value
Pertussis cases	2.860	0.999	D (pertussis cases)	− 2.325	0.020
AP	− 0.568	0.470	D (AP)	− 10.878	< 0.001
AAP	− 0.768	0.382	D (AAP)	− 10.816	< 0.001
AT	− 0.107	0.646	D (AT)	− 14.513	< 0.001
AWV	1.091	0.928	D (AWV)	− 10.286	< 0.001

*AP* aggregate precipitation, *AAP* average air pressure, *AT* average temperature, *AWV* average wind velocity.

**Table 6:** Cointegration test statistics among variables.

Dependent	tau-statistic	*p*-value	z-statistic	*p*-value
D (pertussis cases)	− 7.353	< 0.001	− 109.664	< 0.001
D (AP)	− 22.024	< 0.001	− 260.820	< 0.001
D (AAP)	− 18.570	< 0.001	− 235.248	< 0.001
D (AT)	− 6.739	< 0.001	− 89.174	< 0.001
D (AWV)	− 19.799	< 0.001	− 245.728	< 0.001

*AP* aggregate precipitation, *AAP* average air pressure, *AT* average temperature, *AWV* average wind velocity.

## Discussion

In recent years, pertussis has become a major public health issue due to its persistent re-emerging risk in highly vaccinated populations in China and globally. Basic to any formulation of prevention and control planning is the understanding of its potential influencing factors. Meteorological parameters have been demonstrated to play a significant role in the transmission of contagious diseases, and yet evidence on the long-term quantitative influence of the common monthly meteorological parameters on pertussis is scarce. As far as we know, reported herein is the only research to conduct a comprehensive investigation regarding the long-run effects of variations in monthly weather parameters on pertussis using the data from 2004 through 2018 in Chongqing. The results of this investigation revealed that the reported pertussis cases displayed a marked upward trend since 2014 and had notable seasonal behaviors with a peak occurring in March to August every year and a trough in September until February of the next year. Furthermore, weather variability (particularly temperature, precipitation, air pressure, and wind velocity) with 0–2 month delays can be considered to be important predictors for the spread of pertussis. Such a lead time is of great value for forecasting the epidemic trends of pertussis and thus giving relevant health sectors sufficient time to develop targeted prevention and control planning, and to conduct health education and public health interventions^27^. However, we found that the fit was poor in 2016 (Fig. 4), The plausible explanations may be that the occurrence of pertussis is affected by many factors, such as passive smoke exposure, environmental degradation, population density, and host susceptibility^40–43^, whereas our study failed to include these potential drivers in our regression model, and thus an association cannot be wholly excluded without further investigation of these factors behind the low fit of our model. In addition, the hunt for exact explanations for the low fit of our model goes on. The exact reasons why the rapid increase in the notified numbers of pertussis in recent years failed to be completely clarified in Chongqing; however, several plausible factors may drive such a rise. First, the use of advanced diagnostic methods with higher sensitivity such as simultaneous amplification and testing (SAT), real-time PCR and serologic has improved the recognition of some mildly or moderately infected individuals, especially for people without typical clinical symptoms^4,44^. Second, the medical professionals have enhanced their awareness of the diagnosis and report of pertussis in that this disease was designated as a notifiable infectious disease in China^3,12^. Third, the traditional diphtheria, tetanus and whole-cell pertussis vaccine (DTwP) has been replaced by the combined diphtheria, tetanus and acellular pertussis vaccine (DTaP) in 2010, and yet there may be different efficacies against pathogenic infection between them, this may result in the adaptation or waning after vaccination to the natural or vaccine immunity^12,45,46^. It has been demonstrated that there is a protective effect of pertussis vaccine during the period of around 4–12 years^47,48^. According to the immunization schedule of pertussis in China^11^, it appeared that this situation accounted well for the increased numbers in adolescents and adults in temporality over the last years^44^. Fourth, previous publication has found that young infants accounted for the large proportion of the infected people in Chongqing^12,44^, this may be due to the fact that most young infants at the year of below 3 months were not vaccinated the DTwP vaccination, and the rapid decay of the antibodies to pertussis toxin (PT) and filamentous haemagglutinin (FHA) in infant sera derived from their mothers make infants susceptible to *B. pertussis*^49,50^. Fifth, prior studies reported that new *B. pertussis* strains different from that included in the present vaccines have emerged^47,51^, which may have effect on the manifest rebound of pertussis infections. Sixth, a recent research found that the proportion of the reported pertussis cases aged 1–5 and over 5 years have increased about 4 folds and the proportion aged 3–12 months reduced from 70.59 to 58.39% from 2013 to 2018, respectively, in Chongqing, which can be ascribed to the unqualified vaccine quality^44,52^. Finally, weather parameters may also play an important role in the manifest rebound of pertussis infections, as evidenced by the findings of our study and others^14,24–26^, and it can also be seen from the data in Table S4 that the monthly aggregate precipitation and average wind velocity showed a higher level during the period 2016–2018 as compared to that during the period 2004–2015, while the monthly average air pressure displayed a lower level during the period 2016–2018 relative to the level during the period 2004–2015.

The seasonal profile of pertussis is of great help in understanding the influence of climatic factors on this illness. In this work, pertussis has been identified as being a seasonal disease with spring and summer being thought of as high-risk seasons. Such a seasonal distribution agrees relatively well with the findings from other reports in mainland China^3^, the United States^53^, Italy^54^, the Netherlands^55^, and Australia^56^. However, such a seasonal profile fails to be in good agreement with the finding from research in the south and southeast regions of Brazil, which suggested contrary peak and trough activities in the pertussis incidence to our results. This may be due to the fact that seasonal patterns of pertussis vary widely between countries and regions, despite unclear reasons; possibly climatic drivers have played a role.

In this work, a key finding was that the monthly average wind velocity was the most important determinant (IRR = 3.812, 95% CI 1.243–11.690) in the transmission of pertussis in Chongqing. Current literature exhibits that wind velocity is of significantly positive relevance to the transmission of human brucellosis (in Heibei province)^22^, mumps (in Guangzhou)^29^, and scarlet fever (in Guangzhou)^17^. These conclusions supported our findings to a certain extent. Wind has a great influence on the dilution of the concentration of microbes and on the survival of microbes^57^, and can resuspend bacteria from soil or plant surfaces^58,59^. Considering that the main terrain is mountainous and the wind is often mild in most of the months in Chongqing. When *B. pertussis* stays at the destination before they die, the faster the wind speed becomes, the less time the pathogen remains^57^. That is, faster wind speed will make the concentration analogous to the source concentration of the infection^60^. On the other hand, the liquid on the surface of pathogen that can moisten the surface of pathogenic bacteria and can bond into larger particles, which can further increase surface friction wind speed of pathogenic bacteria, and therefore can inhibit the dispersion of pathogenic bacteria^61^. Whereas faster wind speed may cause more evaporation of liquid on the surface of pathogen and thus the wind speed may have an indirect impact on pertussis by evaporation^22^. Moreover, as a respiratory disease, inhalation of contaminated aerosols also acts as an important route of infection for pertussis^62^. While faster wind speed may accelerate the spread of the contaminated aerosols, and which can also contaminate food or water sources^63,64^.

Temperature is another important driver (IRR = 1.195, 95% CI 1.024–1.395) for the pertussis infection in this work. Temperature has been shown to be in relation to many contagious diseases, such as scarlet fever^65^, HFRS^20^, bacillary dysentery^21^, and human brucellosis^22^. Similarly, in the present work, we found that the monthly average temperature is positively correlated with the number of pertussis cases, which can also be used to explain the peak phenomenon of pertussis incidence observed in hot weather in our study and previous other studies^53,66^. About the positive correlation between them, this is congruous with the recent findings from the studies in Jinan^14,26^ and Auckland^67^, which found that temperature was positively associated with pertussis among different age groups and could be considered as a good predictor. Also, in line with the findings that have been observed in other infectious diseases, such as HFMD (in Guangdong)^68^, bacillary dysentery (in Hunan Province)^31^, and scarlet fever (in Hefei City)^69^. Prior work discovered that extreme heat was positively linked to respiratory diseases^70^, and the patients visiting respiratory emergency department significantly increased during hot weather^71^. These findings further supported our results. But, our finding is inconsistent with prior findings from studies to explore the relationship between other contagious diseases, such as scarlet fever (in Hong Kong)^65^, human brucellosis (in Hubei province)^22^, and HFRS (in Guangzhou and in Chongqing)^20,72^, and climatic factors. This discrepancy may mainly be owing to the various climatic characteristics of the study regions, along with different methods used to investigate the relevance in the studies. And the plausible explanations for the positive correlation between temperature and pertussis may be ascribed to the climatic characteristics in Chongqing. Only when the average temperature is in close proximity to the appropriate temperatures for pathogens will the reported pertussis cases rise, and in this case, the activity of the *adenylate cyclase toxin* is also activated, which has also been shown to be associated with the pathogenesis of pertussis^73^. A study has suggested that *B. pertussis* adenylate cyclase can be activated by the host factor (namely, calmodulin). In the presence of calmodulin, the enzymatic activity is gradually activated as the temperature rises, and the temperature optimum for enzymatic activity is around 35 °C, and the virulence factors can be stimulated by host factors at physiological temperatures and the production of the virulence factors associated with pertussis infections can also be increased at or near these physiological temperatures^73^. Chongqing belongs to a subtropical monsoon humid climate with the temperature ranging from 3.9 °C to 32.4 °C (annual average temperature is 18.694 °C), such environment conditions is adapted for the survival and growth of the pathogens, and thus favoring the transmission of pertussis.

Our results demonstrated that the monthly aggregate precipitation had significantly weak positive contribution to the pertussis cases, with 3.641% (95% CI 0.960–6.330%) rise per 10 mm increment. Such a finding can also be used to account for the peak of pertussis transmission in Chongqing, where the rainy season mostly occurs in spring and summer. Also, a work in Thailand observed that both the pertussis incidence and heavy rainfall peaked in May and June, which means that the precipitation may drive the seasonal behaviors of pertussis^25^. However, our finding is in disagreement with a previous work, although which indicated a positive association between precipitation and pertussis in Jinan using correlation analysis, the authors found that precipitation failed to be a significant predictor in the time series forecasting of pertussis incidence. As for such a discrepancy, this is largely due to the fact that temperature and rainfall were only included in that study^14^. Currently, many water-borne pathogens and vector-borne infectious diseases have been found to be positively linked to precipitation, such as dengue fever^19^, bacillary dysentery^31^, HFRS^72^, etc., which because rainfall can mainly impact such diseases via affecting the development of pathogens and the vectors/hosts^74^. However, there is a current scarcity of relative study and biological evidence to clarify the relationship between rainfall and respiratory diseases, albeit several studies exhibited that precipitation may be associated with scarlet fever^17,75^ and mumps^57^, their findings are contradictory. We surmise that the effect of rainfall on pertussis may not exert a direct influence on pertussis infections but change human activity indirectly as rainy weather limits outdoor activities and work, indoor crowding may facilitate the transmission among susceptible population^76^.

In the current research, the average air pressure was inversely correlated with the pertussis transmission (IRR = 0.964, 95% CI 0.937–0.993). Considering that little literature is available on the association between air pressure and pertussis, the present finding was compared with other respiratory infectious diseases or similar studies. A prior work that used an autoregressive integrated moving average model to investigate the roles of climatic variation in the brucellosis morbidity suggested that air pressure at a lag of two months was negatively associated with brucellosis (β = -0.004, *p* = 0.037)^22^; another studies to explore the effects of weather parameters on mumps in Guangzhou and Fujian indicated that air pressure helped in the spread of mumps^57,77^, these results showed a consistency with our conclusion. While there was work that indicated no role of atmospheric pressure in the scarlet fever incidence in Beijing and Hong Kong^65^. There seems some biological evidence to support such a positive finding. The elevated air pressure may reflect the entry of air conditioning and the improvement in air diffusion conditions^17^, which helps the release and transportation of causative agents, thus leading to a reduced bacterial abundance within the atmosphere. Besides, as discussed above, atmospheric pressure may be likely to indirectly affect pertussis through temperature and precipitation^22^. Previous study reported that under high temperature, the air near the ground can be compelled to move upward, which can in turn lower air pressure in local areas^78^. A low-pressure area tends to rain more often than not^22^. In this case, indoor contact between susceptible populations may facilitate the pertussis infections.

Current evidence shows that the sunshine duration played a positive role in the incidences of scarlet fever (in Beijing)^65^ and mumps (in Fujian province and Jining)^63,77^; whereas we observed no significant effect of the monthly sunshine duration on pertussis. This may due to the climatic characteristics in Chongqing, with the least sunshine and the weakest solar radiation in China. In addition, other studies also reported that there was a positive association between relative humidity and other respiratory infectious diseases, including scarlet fever^69^ and mumps^57^, which is not in accord with our current finding. Hence, further verifying researches into the relationship between weather variability and pertussis should go on in other areas.

This study was focused on the investigation into the effect of variations in monthly meteorological parameters on pertussis infections. As emphasized in prior literature, when performing time series analysis, it is required to consider several issues, including changes in immune persons, strong autocorrelations, a series of possible delay and association patterns, seasonality, long-term trend, and large over-dispersion^27,79^. In this study, we have well taken these issues into account apart from the changes in immune persons due to the lack of the data available. As such, we believe that we have presented accurate and reliable findings. However, several drawbacks should be considered when interpreting these findings. First, under-estimation is an inherent weakness for the passive monitoring system of infectious diseases, though pertussis is a mandatory reporting of infectious disease in China. Second, this work was an ecological study, and thus which fails to allow for investigating individual-based association and has a limited ability to infer the causal association. Third, we cannot obtain the detailed information on the pertussis cases (e.g., sex and age) due to their unavailability, which precludes further stratified analysis. Finally, we failed to incorporate other potential confounding variables into our regression model, owing to the unavailability of monthly data, such as geographic and socioeconomic variables, population density, and host susceptibility.

## Conclusions

Taken together, our work indicated that monthly temperature, precipitation, air pressure and wind velocity played an important role in the transmission of pertussis. These findings offered an insight into the understanding of the epidemic trends in the pertussis incidence, and thus which will be of practical significance for establishing an early warning system and which also hints that it is necessary to integrate climatic factors into public health prevention and control planning of pertussis, especially in the context of global climate change.

## Supplementary information

Supplementary Information.

## Data Availability

All data were presented in our analytical results or please contact the corresponding author on reasonable request.
