# Supplementary material for: The long-term effects of meteorological parameters on pertussis infections in Chongqing, China, 2004–2018
Source: Sci Rep. 2020 Oct 14;10:17235. doi: 10.1038/s41598-020-74363-8 (PMC7560825; doi:10.1038/s41598-020-74363-8)
Supplement: Supplementary file 1 — Supplementary Information. [file 41598_2020_74363_MOESM1_ESM.docx]

Supplementary information: The long-term effects of meteorological parameters on pertussis infections in Chongqing, China, 2004-2018

Yongbin Wang^1,^*, Chunjie Xu^2,^*, Jingchao Ren^1^, Yingzheng Zhao^1^, Yuchun Li^1^, Lei Wang^3^, Sanqiao Yao^1^

^1^ Department of Epidemiology and Health Statistics, School of Public Health, Xinxiang Medical University, Xinxiang, Henan Province, P.R. China

^2^ Department of Occupational and Environmental Health, School of Public Health, Capital Medical University, Beijing, P.R. China

^3^ Center for Musculoskeletal Surgery, Charité–Universitätsmedizin Berlin, Corporate Member of Freie Universität Berlin, Humboldt-Universität Zu Berlin and Berlin Institute of Health, Berlin, Germany

Correspondence: Yongbin Wang ([wybwho@163.com](mailto:wybwho@163.com)), Department of Epidemiology and Health Statistics, School of Public Health, Xinxiang Medical University, Xinxiang 453000, Henan Province, P.R. China ;Tel +86 0373 383 1646

*These authors contributed equally to this work

**Figure S1** The comparison of the four highest annualized incidence loci of pertussis in China from 2004 to 2018.

**Figure S2** The reported monthly pertussis cases in Chongqing, China, 2004-2018.

**Figure S3** The monthly average relative humidity in Chongqing, China, 2004-2018.

**Figure S4** The monthly aggregate precipitation in Chongqing, China, 2004-2018.

**Figure S5** The monthly average air pressure in Chongqing, China, 2004-2018.

**Figure S6** The monthly aggregate sunshine hours in Chongqing, China, 2004-2018.

**Figure S7** The monthly average temperature in Chongqing, China, 2004-2018.

**Figure S8** The monthly average wind velocity in Chongqing, China, 2004-2018.


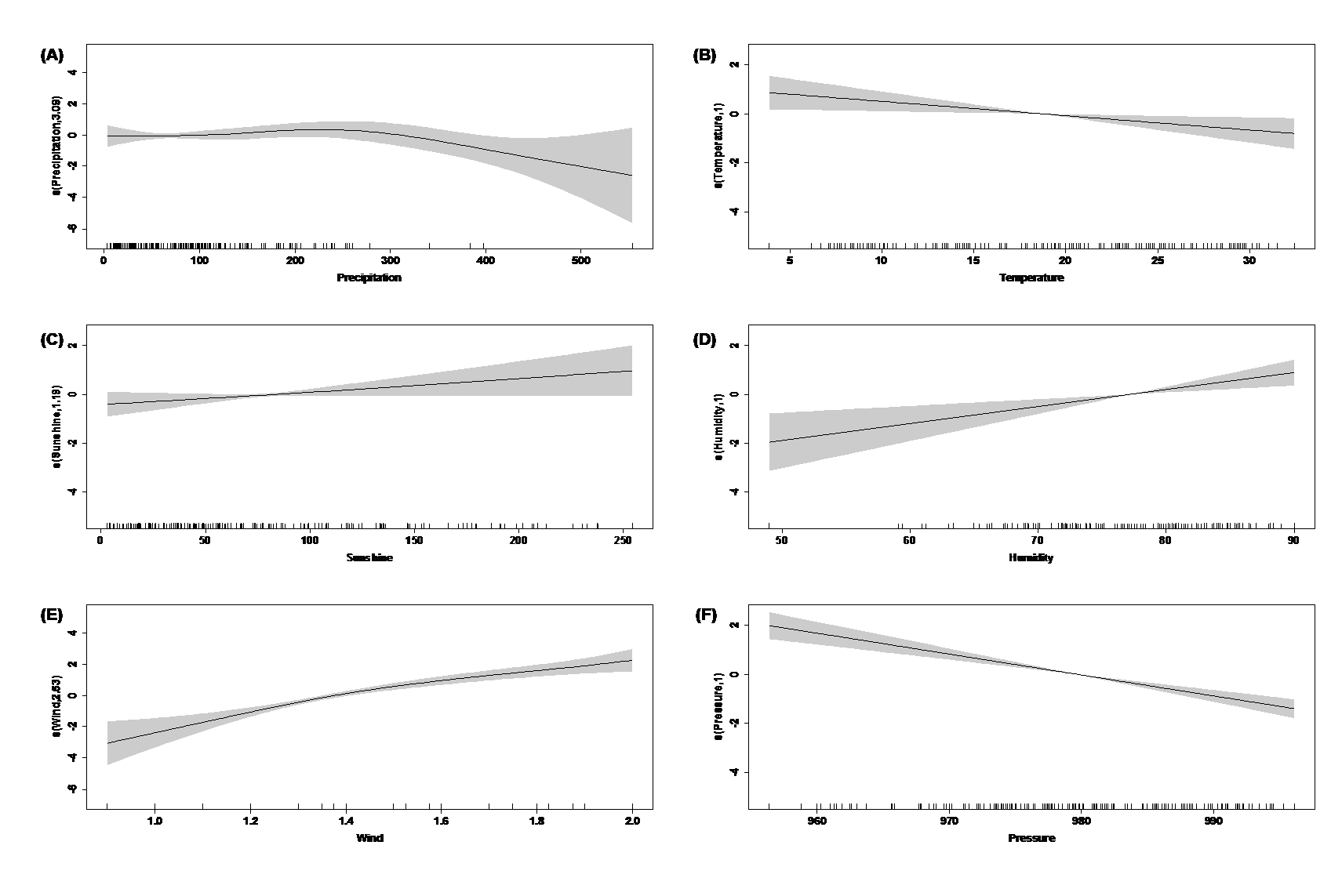


**Figure S9** The estimated smoothness effects of six weather parameters for log(pertussis cases) in the generalized additive model. (A) Aggregate precipitation; (B) Average temperature; (C) Aggregate sunshine hours; (D) Average relative humidity; (E) Average wind velocity; (F) Average air pressure. In the model, the best suitable degree of freedom for every climate parameter was determined based on generalized cross-validation (GCV). It can be seen that the relationship between weather parameters and pertussis cases tends to be linear in Chongqing, China, 2004-2018, so we used a negative binomial multivariable regression to fit our data.

**
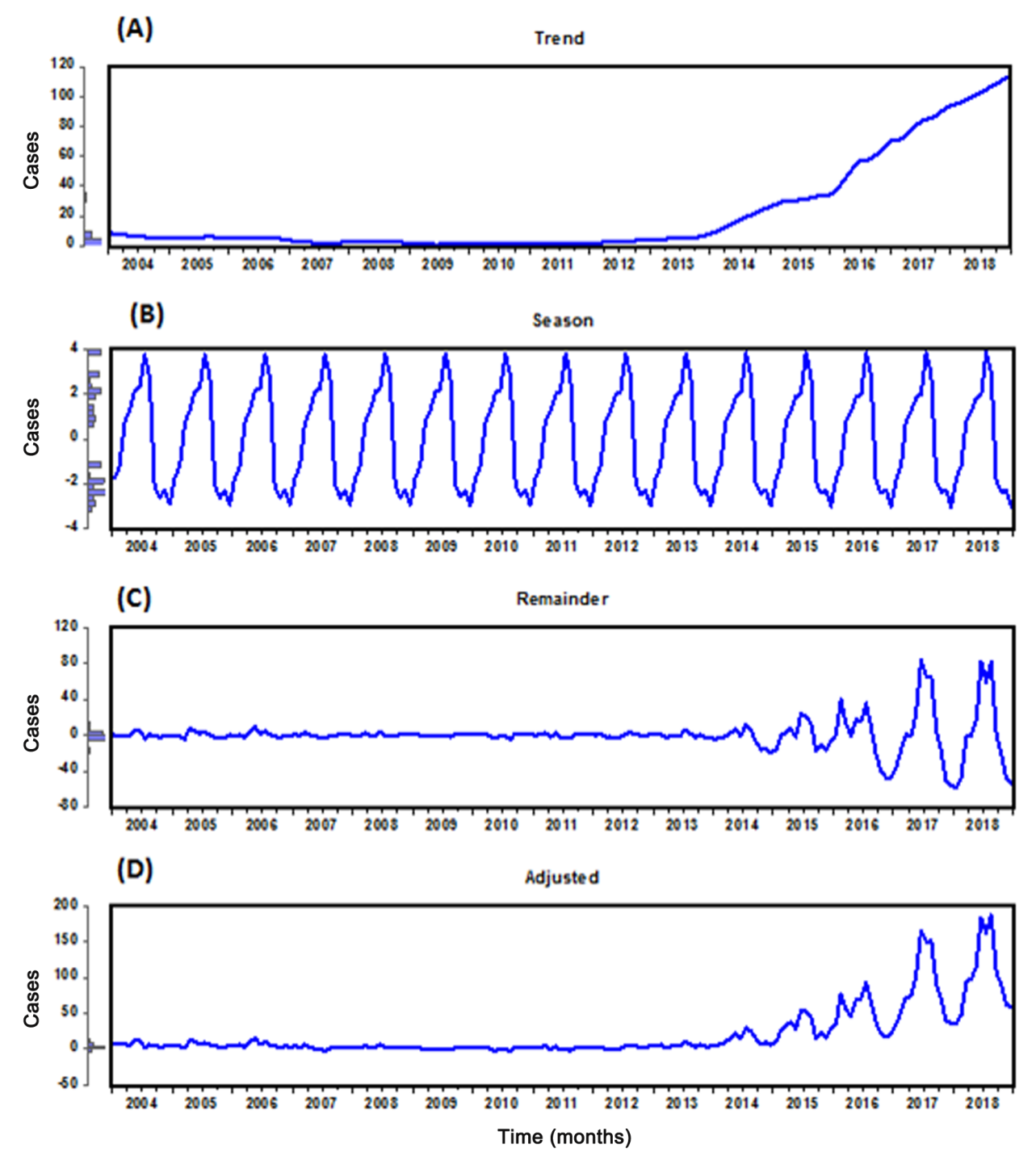
**

**Figure S10** The decomposed trend, seasonal, random, and adjusted components using the Seasonal-Trend decomposition procedure based on Loess method. (A) Time series plot for the trend component; (B) Time series plot the seasonal pattern; (C) Time series plot for the error component; (D) Time series plot for the adjusted pertussis incidence sequence.

**
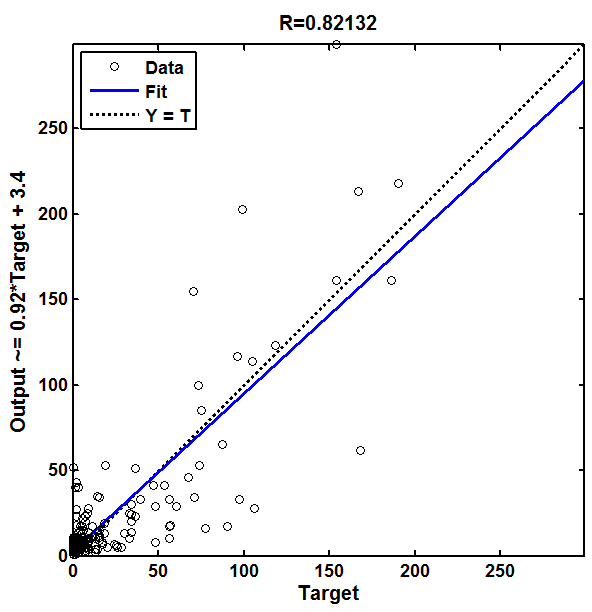
**

**Figure S11** The resulting regression plot displaying the original observations and the predicted values.

| **Parameter** | **Lag0** | | | **lag1** | | | **Lag2** | | |
| --- | --- | --- | --- | --- | --- | --- | --- | --- | --- |
|  | **IRR** | **95% CI** | ***p*-value** | **IRR** | **95% CI** | ***p*-value** | **IRR** | **95% CI** | ***p*-value** |
| AP (mm) | 1.004 | 1.001-1.007 | 0.023 | 1.004 | 1.001-1.007 | 0.008 | 1.004 | 1.000-1.007 | 0.025 |
| AT (˚C) | 1.015 | 0.854-1.206 | 0.868 | 1.085 | 0.918-1.282 | 0.338 | 1.042 | 0.872-1.245 | 0.654 |
| ASH (h) | 1.002 | 0.994-1.010 | 0.633 | 0.999 | 0.991-1.006 | 0.706 | 0.999 | 0.991-1.007 | 0.834 |
| ARH (%) | 1.022 | 0.975-1.071 | 0.374 | 1.013 | 0.968-1.061 | 0.570 | 1.009 | 0.962-1.059 | 0.715 |
| AWV (m/s) | 11.280 | 3.444-36.950 | <0.001 | 10.904 | 3.433-34.641 | <0.001 | 18.179 | 5.305-62.292 | <0.001 |
| AAP (hPa) | 0.895 | 0.871-0.919 | <0.001 | 0.890 | 0.867-0.914 | <0.001 | 0.903 | 0.878-0.929 | <0.001 |
| Year | 1.054 | 1.040-1.069 | <0.001 | 1.056 | 1.042-1.071 | <0.001 | 1.049 | 1.034-1.064 | <0.001 |
| @month=1 | 0.942 | 0.374-2.370 | 0.898 | 4.590 | 1.408-14.966 | 0.011 | 2.363 | 0.694-8.053 | 0.169 |
| @month=2 | 1.026 | 0.423-2.489 | 0.955 | 4.553 | 1.134-18.275 | 0.033 | 10.822 | 1.532-76.455 | 0.017 |
| @month=3 | 0.442 | 0.129-1.508 | 0.192 | 3.697 | 1.171-11.668 | 0.026 | 8.519 | 0.928-78.243 | 0.058 |
| @month=4 | 0.312 | 0.049-1.981 | 0.217 | 1.295 | 0.515-3.255 | 0.583 | 5.553 | 0.827-37.275 | 0.078 |
| @month=5 | 0.206 | 0.019-2.245 | 0.195 | 0.598 | 0.187-1.918 | 0.388 | 2.124 | 0.552-8.173 | 0.273 |
| @month=6 | 0.149 | 0.008-2.897 | 0.209 | 0.305 | 0.063-1.488 | 0.142 | 1.308 | 0.490-3.491 | 0.591 |
| @month=7 | 0.097 | 0.003-2.854 | 0.176 | 0.164 | 0.020-1.329 | 0.090 | 0.860 | 0.287-2.581 | 0.788 |
| @month=8 | 0.120 | 0.004-3.247 | 0.207 | 0.082 | 0.007-1.007 | 0.051 | 0.433 | 0.097-1.935 | 0.273 |
| @month=9 | 0.103 | 0.007-1.513 | 0.097 | 0.043 | 0.004-0.490 | 0.011 | 0.083 | 0.012-0.574 | 0.012 |
| @month=10 | 0.344 | 0.051-2.333 | 0.274 | 0.080 | 0.013-0.505 | 0.007 | 0.091 | 0.014-0.589 | 0.012 |
| @month=11 | 0.515 | 0.150-1.766 | 0.291 | 0.500 | 0.154-1.621 | 0.248 | 0.189 | 0.052-0.690 | 0.012 |
| Scale^#^ | 3.196 | — | — | 2.992 | — | — | 3.466 | — | — |

**Abbreviations:** IRR incident rate ratio, CI confidence interval, AP Aggregate precipitation, AT Average temperature, ASH Aggregate sunshine hours, ARH Average relative humidity, AWV Average wind velocity, AAP Average air pressure, @month=1, …,@month=11 denotes the specified seasonal dummy variables.

^#^ Computed based on the Pearson chi-square.

**Table S1** Negative binomial regression results of climatic variables correlated with the transmission of pertussis in Chongqing, China, 2004-2018

| **Parameter** | **IRR** | **95% CI** | ***p*-value** |
| --- | --- | --- | --- |
| AP (mm) ^*^, 0-month lag | 1.036 | 1.010-1.065 | 0.008 |
| AT (˚C), 0-month lag | 1.195 | 1.024-1.395 | 0.024 |
| ASH (h), 0-month lag | 0.999 | 0.992-1.006 | 0.812 |
| ARH (%), 0-month lag | 1.008 | 0.968-1.050 | 0.702 |
| AWV (m/s), 0-month lag | 3.812 | 1.243-11.690 | 0.019 |
| AAP (hPa), 1-month lag | 0.964 | 0.937-0.993 | 0.014 |
| Pertussis cases, 2-month lag | 1.028 | 1.022-1.034 | <0.001 |
| Year | 1.016 | 1.002-1.031 | 0.023 |
| @month=1 | 2.692 | 1.223-5.923 | 0.014 |
| @month=2 | 2.658 | 1.260-5.605 | 0.010 |
| @month=3 | 0.893 | 0.325-2.456 | 0.827 |
| @month=4 | 0.26 | 0.053-1.269 | 0.096 |
| @month=5 | 0.126 | 0.016-0.992 | 0.049 |
| @month=6 | 0.06 | 0.005-0.794 | 0.033 |
| @month=7 | 0.03 | 0.002-0.576 | 0.020 |
| @month=8 | 0.022 | 0.001-0.417 | 0.011 |
| @month=9 | 0.012 | 0.001-0.141 | <0.001 |
| @month=10 | 0.042 | 0.007-0.245 | <0.001 |
| @month=11 | 0.291 | 0.098-0.857 | 0.025 |
| Scale^#^ | 2.243 | — | — |

**Abbreviations:** IRR incident rate ratio, CI confidence interval, AP Aggregate precipitation, AT Average temperature, ASH Aggregate sunshine hours, ARH Average relative humidity, AWV Average wind velocity, AAP Average air pressure, @month=1, …,@month=11 denotes the specified seasonal dummy variables.

* The effect of per 10 mm increment of aggregate precipitation on pertussis.

^#^ Computed based on the Pearson chi-square.

**Table S2** Estimated effects of meteorological parameters on pertussis morbidity by the final negative binomial regression in Chongqing, China, 2004-2018

| **Parameter** | **IRR** | **95% CI** | ***p*-value** |
| --- | --- | --- | --- |
| AP (mm) ^*^, 2-month moving average lag | 1.098 | 1.050-1.146 | <0.001 |
| AT (˚C) , 2-month moving average lag | 1.232 | 1.004-1.511 | 0.046 |
| ASH (h) , 2-month moving average lag | 0.997 | 0.990-1.003 | 0.324 |
| ARH (%), 2-month moving average lag | 1.005 | 0.963-1.049 | 0.809 |
| AWV (m/s) , 2-month moving average lag | 7.572 | 1.830-31.333 | 0.005 |
| AAP (hPa) , 2-month moving average lag | 0.952 | 0.923-0.982 | 0.002 |
| Pertussis cases, 2-month lag | 1.022 | 1.016-1.029 | <0.001 |
| Year | 1.022 | 1.007-1.037 | 0.004 |
| @month=1 | 4.356 | 1.461-12.988 | 0.008 |
| @month=2 | 9.710 | 2.498-37.745 | 0.001 |
| @month=3 | 6.288 | 2.112-18.717 | 0.001 |
| @month=4 | 1.577 | 0.681-3.655 | 0.288 |
| @month=5 | 0.364 | 0.105-1.259 | 0.110 |
| @month=6 | 0.092 | 0.014-0.625 | 0.015 |
| @month=7 | 0.039 | 0.003-0.500 | 0.013 |
| @month=8 | 0.014 | 0.001-0.271 | 0.005 |
| @month=9 | 0.005 | 0.000-0.084 | <0.001 |
| @month=10 | 0.013 | 0.001-0.118 | <0.001 |
| @month=11 | 0.151 | 0.041-0.556 | 0.004 |
| Scale^#^ | 2.071 | — | — |

**Abbreviations:** IRR incident rate ratio, CI confidence interval, AP Aggregate precipitation, AT Average temperature, ASH Aggregate sunshine hours, ARH Average relative humidity, AWV Average wind velocity, AAP Average air pressure.

* The effect of per 10 mm increment of aggregate precipitation on pertussis, @month=1, …,@month=11 denotes the specified seasonal dummy variables.

^#^ Computed based on the Pearson chi-square

**Table S3** Estimated effects of two-month moving averaged meteorological parameters on pertussis morbidity by the negative binomial regression in Chongqing, China, 2004-2018.

| **Period** | **Variable** | **Mean** | **S.D.** | **Min** | **P_25_** | **P_50_** | **P_75_** | **Max** |
| --- | --- | --- | --- | --- | --- | --- | --- | --- |
| 2004-2015 | AP (mm) | 95.381 | 84.745 | 3.400 | 30.800 | 77.300 | 121.550 | 553.400 |
|  | AT (˚C) | 18.776 | 7.308 | 3.900 | 12.825 | 19.500 | 24.775 | 32.400 |
|  | ASH (h) | 79.084 | 60.856 | 0.000 | 28.275 | 63.550 | 118.825 | 254.300 |
|  | ARH (%) | 76.897 | 7.348 | 49.000 | 72.275 | 78.000 | 82.300 | 90.000 |
|  | AWV (m/s) | 1.351 | 0.174 | 0.900 | 1.200 | 1.400 | 1.500 | 1.900 |
|  | AAP (hPa) | 981.991 | 8.199 | 956.400 | 975.175 | 981.950 | 988.700 | 996.100 |
| 2016-2018 | AP (mm) | 101.953 | 75.689 | 9.500 | 34.743 | 98.750 | 142.088 | 397.475 |
|  | AT (˚C) | 18.365 | 7.560 | 6.700 | 10.713 | 18.738 | 23.900 | 30.400 |
|  | ASH (h) | 92.819 | 68.339 | 14.600 | 39.275 | 70.738 | 133.194 | 238.000 |
|  | ARH (%) | 78.704 | 6.484 | 65.000 | 74.200 | 80.300 | 84.075 | 88.400 |
|  | AWV (m/s) | 1.624 | 0.193 | 1.350 | 1.425 | 1.600 | 1.800 | 2.000 |
|  | AAP (hPa) | 971.052 | 7.066 | 985.800 | 964.175 | 970.550 | 977.700 | 981.600 |

**Abbreviation:** AP Aggregate precipitation, AT Average temperature, ASH Aggregate sunshine hours, ARH Average relative humidity, AWV Average wind velocity, AAP Average air pressure, S.D. Standard deviation.

**Table S4** Summary statistics for the monthly weather parameters during different periods in Chongqing, China, 2004-2018
